# Practical Use of Metal Oxide Semiconductor Gas Sensors for Measuring Nitrogen Dioxide and Ozone in Urban Environments

**DOI:** 10.3390/s17071653

**Published:** 2017-07-19

**Authors:** Philip J. D. Peterson, Amrita Aujla, Kirsty H. Grant, Alex G. Brundle, Martin R. Thompson, Josh Vande Hey, Roland J. Leigh

**Affiliations:** 1 Department of Physics and Astronomy, University of Leicester, Leicester LE17RH, UK; pp228@le.ac.uk (P.J.D.P); aa946@le.ac.uk (A.A.); kirsty.h.g.1001@gmail.com (K.H.G.); agbrundle@hotmail.co.uk (A.G.B.); martinrt38@gmail.com (M.R.T.); jvh7@le.ac.uk (J.V.H.); 2 Earthsense Systems Ltd., Leicester LE45NU, UK

**Keywords:** metal oxide, gas sensors, environmental monitoring

## Abstract

The potential of inexpensive Metal Oxide Semiconductor (MOS) gas sensors to be used for urban air quality monitoring has been the topic of increasing interest in the last decade. This paper discusses some of the lessons of three years of experience working with such sensors on a novel instrument platform (Small Open General purpose Sensor (SOGS)) in the measurement of atmospheric nitrogen dioxide and ozone concentrations. Analytic methods for increasing long-term accuracy of measurements are discussed, which permit nitrogen dioxide measurements with 95% confidence intervals of 20.0 $\mu g\;m^{-3}$ and ozone precision of 26.8 $\mu g\;m^{-3}$, for measurements over a period one month away from calibration, averaged over 18 months of such calibrations. Beyond four months from calibration, sensor drift becomes significant, and accuracy is significantly reduced. Successful calibration schemes are discussed with the use of controlled artificial atmospheres complementing deployment on a reference weather station exposed to the elements. Manufacturing variation in the attributes of individual sensors are examined, an experiment possible due to the instrument being equipped with pairs of sensors of the same kind. Good repeatability (better than 0.7 correlation) between individual sensor elements is shown. The results from sensors that used fans to push air past an internal sensor element are compared with mounting the sensors on the outside of the enclosure, the latter design increasing effective integration time to more than a day. Finally, possible paths forward are suggested for improving the reliability of this promising sensor technology for measuring pollution in an urban environment.

## 1. Introduction

Human activity has affected the nitrogen cycle through nitrogen fertilization, biomass burning and fossil fuel combustion, and this has led to enhanced levels of NO_2_ in the atmosphere [1,2]. Annual mean concentrations of NO_2_ are highest in urban environments and around major highways [3]. With an increasing percentage of the world’s population living in cities, an understanding of local pollution variation is essential in improving public health. Nitrogen oxides in the atmosphere are harmful when inhaled, but also play many important roles in the biosphere; they are involved in the formation of ozone, acidification and eutrophication of soil, groundwater and surface waters and can decrease ecosystem vitality and biodiversity, and cause groundwater pollution [1,4].

Nitrogen oxides exist in a rapid equilibrium system with ozone (the Leighton relationship), and their production and abundance are dependant on both temperature and ultraviolet radiation. The variation of both of these factors, on the scale of seconds and meters, results in the variation of urban air pollution being greater within cities than between them [5,6,7]. Consequently, high temporal and spatial resolution instruments are required to fully understand nitrogen dioxide behaviour in urban environments.

While satellite instruments such as the OMI (Ozone Monitoring Instrument) have been used to map pollutants on a regional scale, OMI’s once-daily global coverage, with a minimum pixel size of 13 km × 24 km is far too broad for urban measurements [8]. An alternative to space-based instruments is aerial surveys, such as ANDI (Atmospheric Nitrogen Dioxide Imager) [9], which produce maps with resolutions of a few dozen meters per pixel. However, flights are isolated and do not provide the daily constant monitoring of a satellite mission. Alternatively, ground based measurements provide data from discrete locations, but are capable of high temporal resolution. The AURN (Automatic Urban and Rural Network) stations in the U.K. operated by DEFRA (Department for the Environment, Food and Rural Affairs) contain sophisticated reference instruments [10] that precisely measure atmospheric composition and provide public reports of it on an hourly basis. In order to understand important fluctuations on shorter time scales, higher temporal resolution is needed. Furthermore, the spatial resolution of the stations is limited by their high cost (>£10k) and size, making them poorly suited to identifying pollution hotspots. [11]

Dispersion modelling in cities using Gaussian sources or computational fluid dynamics, combined with modelling of the chemical reactions in the atmosphere, can predict concentrations with resolutions as high as programmer ingenuity and supercomputer time will allow, but they must be constrained by in situ measurements to enhance scientific utility.

A network of small air quality sensors with appropriate accuracy can produce a spatially-resolved picture of pollution variations on the urban scale. Since the development of the Palmes diffusion tubes in 1976 [12], it has been possible to take inexpensive local measurements of nitrogen dioxide, but over time scales of a few weeks. Recent electronic sensors have promised both lower costs than satellite experiments and chemiluminescence sensors and higher time resolution than diffusion tubes.

Such devices make use of inexpensive sensing elements that use either electrochemical reactions between target gases and a substrate electrode, producing a signal current in proportion to the gas concentration [13], or a heated Metal Oxide Semiconductor (MOS) element with a conductive region that varies depending on the presence of adsorbed reducing or oxidizing gases on its surface [14]. However, while the unit costs of such sensors may be lower than that of the chemiluminescence instruments used in AURN stations, the network also requires a different level of management and maintenance [15], MOS sensors are known to suffer from worse cross-sensitivity, and low cost devices in general can have design compromises that make their readings less trustworthy.

As precise and small alternatives to monitoring stations, MOS sensors have previously been used in industry as safety devices [16]; of particular importance is H_2_ in the transition to a carbon-low, hydrogen-inclusive energy economy [17,18]. They have also been used in environmental monitoring to detect NO_2_ [19], VOCs [20,21], ozone [22] and CO [23,24]. However, in the face of increasing demand, MOS sensors have significant obstacles to their direct use as air quality monitors. Their output signal is influenced by the concentrations of both the target and interfering gases, as well as the temperature and humidity effects [15,25,26,27]. Inherent variability between sensors, due to the manufacturing process, also makes for weak reproducibility [28]. Furthermore, baseline drift over time, caused by either changes in the heat output of the sensing element or due to poisoning (irreversible bonding) to the the sensor surface, presents a significant problem for deployments that last over 2–3 months [29,30].

In spite of these obstacles, MOS sensors still present an enticing way of measuring NO_2_ and O_3_ concentrations in urban environments. A sensor platform was designed to evaluate their capability and develop them into a more mature product. These were tested extensively in the field, with the resulting performance data informing the design of both the sensor electronics and the casing.

The goal of this research is to produce a NO_2_ and O_3_ sensor with enough accuracy to be useful for urban pollution measurements. The target accuracy at 95% confidence is 20 $\mu g\;m^{-3}$ for both NO_2_ and O_3_. European directives define target and absolute maximum thresholds for the concentration of both these gases [31]. For NO_2_, the maximum average yearly concentration is 40 $\mu g\;m^{-3}$ and hourly concentrations must not exceed 200 $\mu g\;m^{-3}$ more than eighteen times a year. For O_3_, the daily 8-h mean must not exceed 120 $\mu g\;m^{-3}$ more than 25 times over three years. Urban environments frequently experience NO_2_ levels that exceed the yearly target. An accuracy of 20 $\mu g\;m^{-3}$ would provide enough resolution to make these sensors a useful supplement to modelling and satellite campaigns, in the context of these targets.

In addition the response of the MOS sensors themselves, the design of the equipment used to support them has a practical effect on the quality of results, as do calibration techniques, both of which are discussed in this work.

### 1.1. MOS Sensors

The phenomenon of heated metal oxides changing in resistance in response to the composition of the atmosphere around them has been known for over forty years. For example, sensors using a SnO_2_ substrate are sensitive to a spectrum of combustible hydrocarbons and have been used in industrial safety equipment and chromatographs [32]. Other MOS materials have been investigated, including Ga_2_O_3_ and WO_3_, which have different gas sensitivity profiles [33], although as mentioned previously, all such sensors exhibit significant cross-sensitivity.

**Figure 1:**
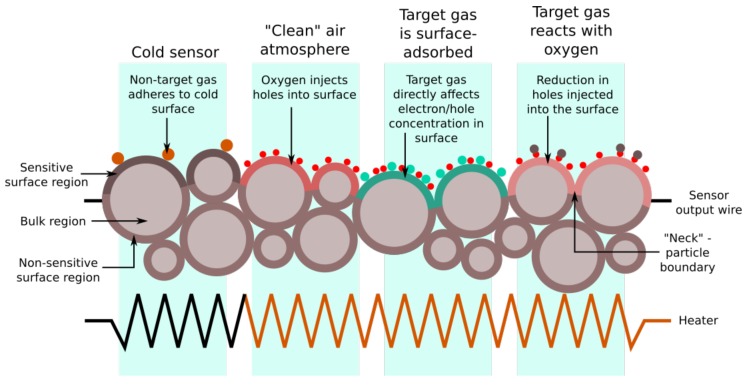
Diagram of the various types of interaction between atmospheric gases and an MOS sensor surface. In the leftmost region, the sensor is unpowered (and exhibits the base resistance). The three other regions of the diagram describe different processes that actually occur simultaneously to varying degrees. The sensor’s output is the resistance across the whole of the sensor material, which forms a resistor network with contributions from both the bulk and surface regions (although the non-sensitive surface will have similar properties to the bulk). This model of the sensor material also explains the wide variation in base resistance between individual sensors of the same type, as the random nature of the surface geometry means an equally random network of resistances. This diagram is a two-dimensional representation of a three-dimensional material; in an actual sensor, the sensitive region is spread into the surface with a distance dependent on the grain size and arrangement resulting from the sintering.

The structure of an MOS gas sensor is shown in Figure 1. The sensor element is typically heated to a few hundred degrees centigrade using a small resistive heater. The equivalent circuit model described by Naisbitt et al. [34] defines three regions of the semiconductor: the surface, which interacts with the gas, the bulk, which is unaffected by it, and the particle boundary, which lies in between these two. The particle boundary is situated at a distance from any material exposed to the atmosphere equal to the Debye length: the distance into the sensor that chemical electrostatic effects can propagate, related to the material’s physical properties. At high temperatures, oxygen atoms bond onto the boundary, extracting electrons in the process from the semiconductor’s surface region. The oxygen either then directly reacts with ambient gases, or these gases also bond onto the sensor, which causes more charge carriers to be withdrawn or injected into the surface region. This changes the sensor resistance. Various types of interaction with the sensor are shown in Figure 1.

The semiconductor type determines the way resistance is affected by gas interactions. For example, oxygen atom reactions will reduce the resistance of a p-type semiconductor where holes are the majority charge carriers, as will other oxidizing gases. Reducing gases will have the opposite effect for a p-type semiconductor. The converse to all of this is true for n-type semiconductors.

The consequence of this model is that the sensor’s response is only partially a function of the amount of gas to which the surface is exposed. Instead, the sensor will have a baseline resistance that is related to the bulk and particle boundary resistance. Because of the random geometry of the granular sensor surface, the baseline resistance will vary between individual sensors. An experiment to examine this variability is shown in Section 3.5.

Any sensor material may participate in reactions with a variety of different gas species beyond the target gas. In ideal conditions, these reactions are reversible and have an equilibrium point determined by the temperature of the sensor; however, some reactions may be irreversible and will poison the sensor, making it less sensitive. The natural consequence of this system of reactions is that although the chemical properties of the sensor material and the temperature of the element might favour reactions with a particular target gas, cross sensitivity with many different species is a unavoidable.

Although the deployment of multiple different sensors can compensate for the cross-sensitivity issues in calibration, it cannot eliminate it. MOS sensors can thus be used only in situations where any interfering species can either be measured by other means, or they must be calibrated regularly and used in locations where the background varies in concentration slowly compared with the target gases.

Modern monolithic MOS sensors have a built-in resistive heater for bringing the element up to optimum temperature, but the actual surface temperature is a function of the power dissipated by the heater and the ambient conditions. The heater resistance reduces over time [15], and this variation has a corresponding effect on the device’s selectivity as the sensor ages. Ambient humidity can also influence the conductivity. Water vapour adsorbed onto the sensing layer can react and act as a reducing gas, mitigating the sensor response to oxidizing gases.

An important practical consideration with any in situ air quality sensor design is ensuring adequate flow of sampling air through the device. Stale air inside a casing will produce unrepresentative results, and even sensors mounted outside a casing might not get a properly-mixed sample. An experiment was performed to investigate this issue, shown in Section 3.4.

### 1.2. Previous Work from Other Groups

MOS siensors have been the subject of active research for some time, and there is a well-established literature on the subject. An example of an academically-developed small sensor is the M-Pod from the University of Boulder, Colorado. First in development from 2009 [19], the M-Pod was designed from the outset to support scientific measurements and has already been deployed as part of measurement campaigns by the university [35]. Equipped with metal oxide sensors, it can return results for both ozone and nitrogen dioxide. Due to its academic background, several conclusions relevant to the use of such sensors have been published [30], including the importance of calibration at a specific location. As shown by Piedrahita et al, signals from nine M-Pod during calibration next to a reference instrument in an urban environment over the course of nine days were fitted to NO_2_ with a 95% confidence of 31.92 $\mu g\;m^{-3}$ and to O_3_ with a 95% confidence of 24.4 $\mu g\;m^{-3}$.

A more varied experiment that compared the raw outputs from different sensor designs over five months is described in [36]. This experiment used an artificial neural network to fit the output from both MOS and electrochemical sensors to a weather station reference in a semi-rural environment and to determine their accuracy. The first week of this period was used as training, and residuals were taken of the rest of the data. The network achieved excellent results for NO_2_, far better than a linear model was capable of, although such a model also proved adequate for O_3_. The results are in a format that is not easily directly compared to the results in this paper, but it must be noted that residuals from both target gasses gradually get worse over a period of roughly two months from the time of calibration. This work seeks to improve this.

### 1.3. Theory of MOS Sensor Response

Converting measurements of the sensor resistance into gas concentrations is non-trivial, as device’s response is non-linear with respect to both target and interfering gases.

**Figure 2:**
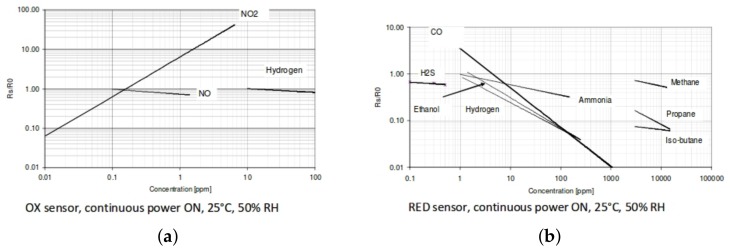
(**a**) MICS-4514 oxidising (OX) gas sensor element nonlinear response to nitrogen dioxide, from the manufacturer’s datasheet [37]; (**b**) MICS-4514 reducing (RED) gas sensor element response to various gases. $R_{s}$ is the sensor’s actual resistance, compared to the base resistance $R_{0}$. Reproduced with permission from SGX Sensortech.

Theoretically, metal oxide sensors change their resistance in response to atmospheric compositions in accordance with Equation (1):

(1) $$R_{s}=R_{b}+R_{g}\sum_{i\in G}f_{i}\left(a_{i}\left(\left[G_{i}\right]\right),T_{a},T_{h}\right)+R_{err}$$

where $G_{i}$ is a specific atmospheric gas and $\left[G_{i}\right]$ its concentration, with $a_{i}$ being a function giving the fractional quantity of the target gas currently adhered to active sites on the semiconductor surface, relative to the entire semiconductor surface. $T_{a}$ is atmospheric temperature; $T_{h}$ is sensor element temperature; the $R_{b}$ and $R_{g}$ terms are base and gas sensitivity resistances to be found; and $R_{err}$ constitutes any error due to the sensor ageing or becoming poisoned. *f* is a non-linear function unique to every gas in the atmosphere. As discussed, the different types of metal oxide sensors will have different responsiveness to different gas species, as shown in Figure 2.

The *a* term is also important, as each sensor element has a limited active surface area, and the equilibrium equations are dynamic processes. Ideally when the sensor is hot, gases cycle fairly frequently between the atmosphere and the surface of the sensor, and this keeps the *a* function approximately proportional to the concentration of each gas. This approximation holds only when the characteristic time scale of every reaction is less than the time scale over which the atmospheric conditions next to the sensor vary. In reality, each equilibrium reaction will have its own rate equation, and some gases may adhere permanently to the sensor surface, which will increase the *a* term for that gas. The sum of all *a* must be less than the active surface area $A_{sensor}$, which means that any gases that latch on to the surface permanently will reduce the device’s sensitivity to anything else.

A possible form for the *f* gas response function is derived in Naisbitt et al. [34] based on the sensor’s physical properties, but the equation can also be inferred through testing the sensors in a closed gas cell. Details of an experiment performed to do this are given in Section 3.1.

### 1.4. Warm-Up Time

An initial warm-up period is required for the sensors to achieve chemical equilibrium with the atmosphere after they are turned on. When the sensor elements are cold, long chain hydrocarbons and other volatile substances deposit onto their surface. When the temperature of the element changes, these larger molecules take time to evaporate. The warm-up time is the period after which changes in sensor response can be attributed solely to changes in atmospheric composition, and in a fixed atmosphere, this time can be measured precisely. A practical balance between precision (longer warm-up time) and time efficiency (shorter warm-up time) should be reached before any sensors can be deployed.

Equation (1) is the pseudo-static equation giving the response of the sensor at a point in time. The *a* terms in that equation are proportional to the actual concentration of gas in the atmosphere only when the rates of the equilibrium reactions are all faster than the rate at which the atmosphere in front of them varies. In situations where the sensor element cools to room temperature, this can no longer be assumed, as the Arrhenius law reminds us that the reaction rate decreases exponentially as temperature falls.

A simple way of modelling the sensor under changing atmospheric conditions involves separately considering the adsorption and desorption of a target gas gas, with the sensor surface area with that particular gas attached to it represented by $a_{gas}$. The rate at which the gas adheres to the sensor surface depends on the concentration of the gas in the region directly above the sensor and the sensor’s active surface area *A*. In the absence of any of these sites being occupied, the fractional rate at which gas attaches to the sensor surface is:

(2) $$\frac{1}{A}\frac{da_{gas}}{dt}=\left[G_{gas}\right]hA\lambda_{a}\left(T\right)$$

where *A* is the total sensor area, $\left[G_{gaS}\right]$ is the gas concentration, *h* is the thickness of the interacting gas layer above the sensor surface (a constant) and $\lambda_{a}$ is a constant representing the probability of gas attachment as a function of temperature *T*, with units of events per second, a function of temperature.

In a real sensor, some proportion of the sensor will be occupied not just by the target gas, but by any other gases present in the atmosphere (*G*). Consequently, the rate of adsorption is reduced as the level of adsorption of any gas increases:

(3) $$\frac{1}{A}\frac{da_{gas}}{dt}=\left[G_{gas}\right]\left(A-\sum_{i\in G}a_{i}\right)\lambda_{a}\left(T\right)$$

Equation (3) only represents attachment. Adding a detachment term proportional to the amount that is currently bonded to the sensor gives:

(4) $$\frac{1}{A}\frac{da_{gas}}{dt}=-\frac{a_{gas}\lambda_{d}\left(T\right)}{A}$$

where $\lambda_{d}$ is a detachment rate constant. The sum of these rates gives the proportional rate at which the sensor area $a_{G}$ occupied by the target gas changes:

(5) $$\frac{1}{A}\frac{da_{gas}}{dt}=\left[G_{gas}\right]h\left(A-\sum_{i\in G}a_{i}\right)\lambda_{a}\left(T\right)-\frac{a_{gas}\lambda_{d}\left(T\right)}{A}$$

From Equation (1), the area already taken up by other gasses is an issue if the interaction lifetime of the gasses with the sensor become significant on the time scale of the experiment; this results in sensor hysteresis and non-zero response time during normal operation.

In a static atmosphere after a long enough period of time, Equation (5) will settle to zero for all gases. Solving this system of partial differential equations can give a baseline level of adhesion that the sensor will gravitate to in a long enough time scale; however, due to the nonlinearity of *f*, this will not help us in practical terms, and the baseline must instead be found experimentally.

However, if power is interrupted, the surface temperature will change, and the λ terms will change with it. Since the form of Equation (5) is a linear sum of proportional terms, it follows that the equation has an exponential form when solved for time:

(6) $$a\left(t\right)=k_{a}e^{(\lambda_{a}-\lambda_{d})t}+k_{c}$$

where the constant $k_{c}$ represents the new baseline, $k_{a}$ is a constant that must be found experimentally and *t* is the time since the power was turned off. This understanding of baselines is based on the unphysical assumption that the temperature will near-instantly change when power is interrupted; in reality, the $k_{c}$ term will change if the temperature of the surface varies.

**Figure 3:**
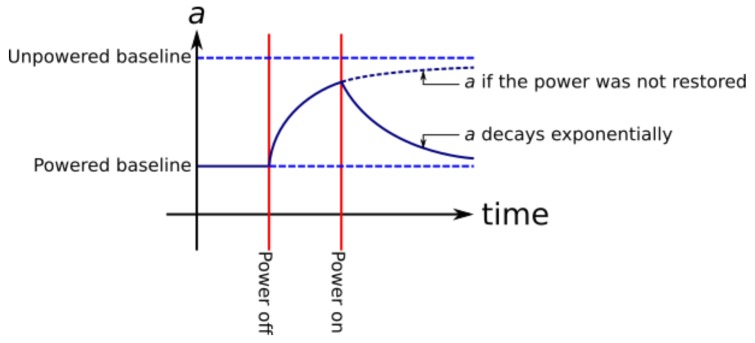
Example curve of net gas adhesion after a power supply interruption event. The *a* term is the amount of sensor surface area bonded to a target gas.

From Equation (6), the power supply interruption episode will have the characteristic form shown in Figure 3. In the field, this curve is of importance because an ideal sensor will always follow the baseline, when the gases adhering to the surface are in equilibrium, and consequently, the output voltage from the sensor is directly related to the concentration of the target gas. When the power supply is cut and restored, the amount of time until the sensor can be said to be functioning normally again is the point at which the contribution to the sensor’s resistance as a result of the power interruption event is minimal compared to other sources of error within the sensor. If the sensor obeys the laws described above, then the amount of time before the sensor is ready for operation will be predictable and will vary with the duration of the power interruption.

An experiment testing this hypothesis is described in Section 3.3.

### 1.5. Statistical Definitions

This paper uses three different statistics to determine the quality of any predicted gas values:

The Residual Standard Error (RSE; Equation (7)) gives a sense of the average of the absolute distance between predicted and observed data,

(7) $$\sigma_{RSE}=2*\sqrt{\sum_{i}^{N}{\left(x_{i}-r_{i}\right)}^{2}}$$

where *x* is a predicted value and *r* is a reference value and gives a range between which 95% of the sensor’s data points lie.

The Fractional Error (FE; Equation (8)) is useful because it is not thrown off by small reference values:

(8) $$\delta_{FE}=\sqrt{\sum_{i}^{N}{\left(\frac{x_{i}-r_{i}}{r_{i}}\right)}^{2}}$$

The Pearson Correlation Coefficient (PCC; Equation (9)) is a measure of how far two variables move together. When it is closer to one, the predicted time series will have the same “shape” as the reference, although the absolute values might be different.

(9) $$\rho \left(x,r\right)=\frac{1}{N-1}\sum_{i}^{N}\frac{x_{i}-\bar{x}}{\sigma_{x}}\frac{r_{i}-\bar{r}}{\sigma_{r}}$$

where $\bar{x}$ is the mean and $\sigma_{x}$ and $\sigma_{r}$ the standard deviation of the predicted and reference values, respectively.

## 2. Materials and Methods

The Small Open General purpose Sensor (SOGS) sensor platform has been in development since 2014, principally as a test bed for MOS sensors, but also as a more versatile instrument platform that could be easily incorporated as a peripheral to larger systems or a datalogger suited to long-term deployments, using a standardized electrical interface. In addition to MOS sensors, the SOGS platform has been deployed with air opacity sensors, optical particle counters and electrochemical gas sensors.

### 2.1. Instrument Architecture

**Figure 4:**
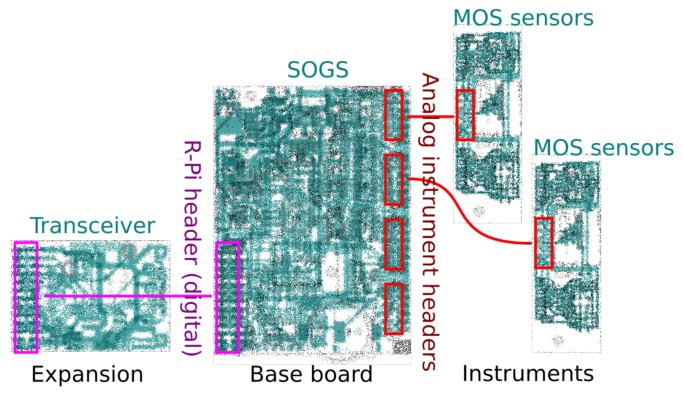
Small Open General purpose Sensor (SOGS) daughterboard example configuration. This diagram shows the base board, an expansion board (through the digital Raspberry Pi header) and instrument boards (through the analogue headers). Multiple boards can be stacked through the digital header, and up to four different analogue instruments can be connected. Each instrument board connector carries power and eight analogue signals.

**Figure 5:**
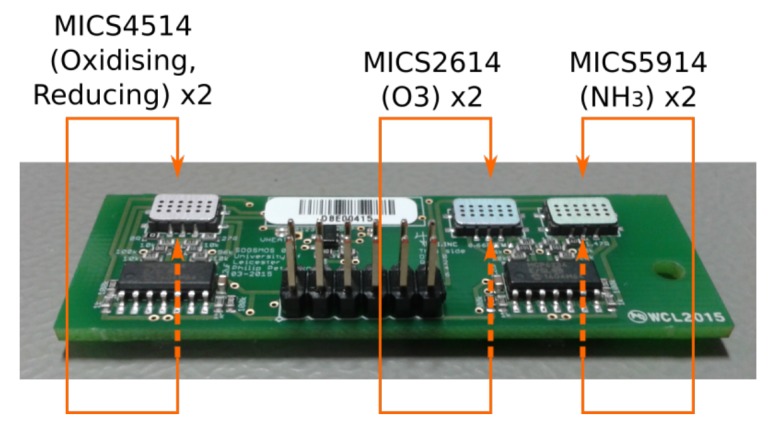
The layout of sensors on an SOGS MOS instrument board. Visible are the side *a* set of sensors; the second set of sensors *b* is on the reverse side. The MICS4514 contains two different sensor elements.

Sensors using SOGS have a common architecture, shown in Figure 4. The SOGS base board is connected to “instrument boards” through either its analogue or digital headers. The MOS instrument board design used to capture the data described in this paper is shown in Figure 5 and carries two sets of four gas sensors (capable of detecting: reducing gases, oxidising gases, ozone and ammonia). The base board is equipped with relative humidity and temperature sensors, the details of which are summarised in Table 1.

The doubling up of each type of sensor allows one set to work as a backup in case a manufacturing defect or sensor corruption over time renders one set unusable. Any two sensors of the same kind tend to be quite strongly correlated with each other (unless one of them is failing), as discussed in Section 3.5.

The SOGS logs voltages from the sensor amplifiers that are directly proportional to the sensor resistance. This is relevant for Section 3.5, in particular for comparing the ratios of typical sensor voltages.

### 2.2. Zephyr

**Figure 6:**
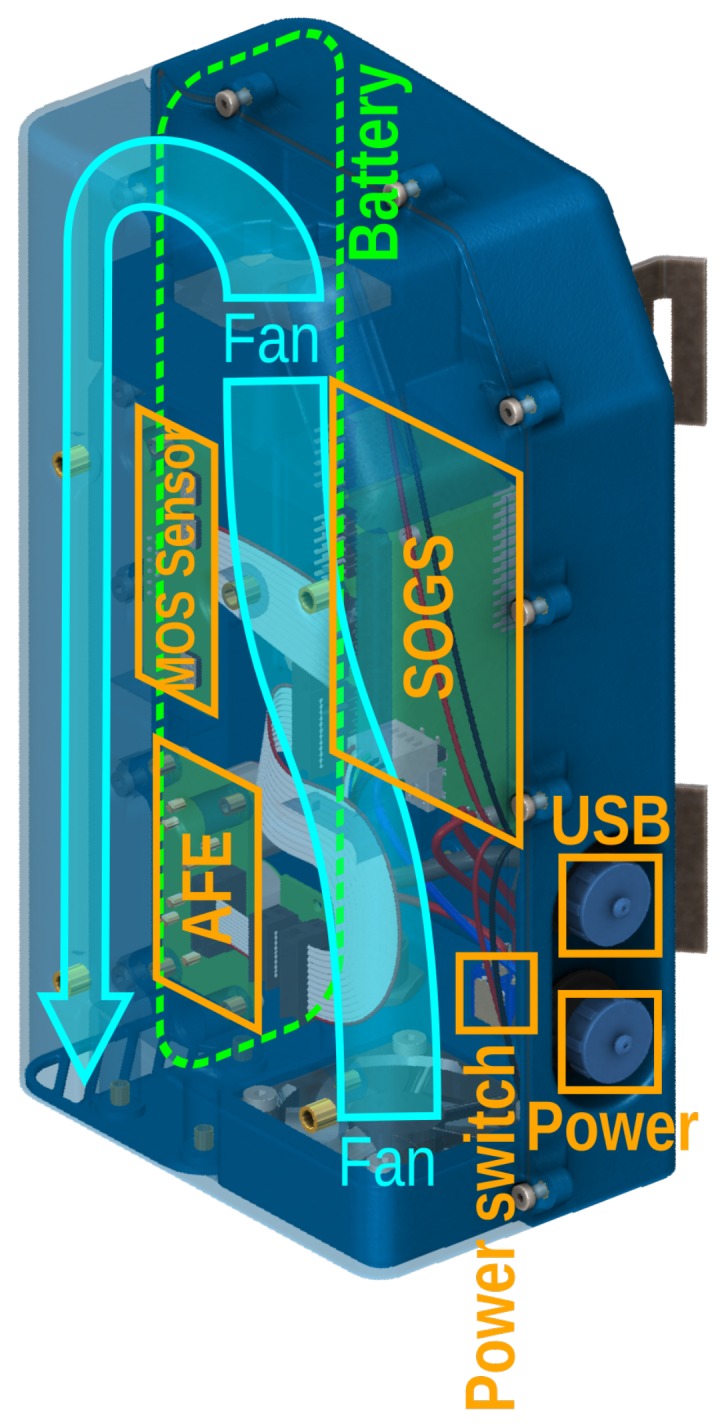
Cutaway of the prototype Zephyr enclosure, showing the layout of vital components and interfaces. Major components are labelled in orange, including an Alphasense AFE electrochemical sensor, which is not the subject of this paper. The MOS instrument boards are suspended away from the casing wall to allow air to flow past both sides.

SOGS is the platform on which the Zephyr air quality monitor is based, prototypes of which were used to acquire the data for this paper. Zephyr consists of a waterproof outdoor casing equipped with an SOGS Figure 6, transceiver and an MOS instrument board. The case has two fans for drawing air across the sensors, which can fully refresh the air inside in less than a second, and can run off a battery that lasts for roughly 12 hours. Computer airflow simulation informed the casing design and ensured that stale air was recirculated instead of being trapped against the sensor elements.

The MOS sensor instrument boards fit onto SOGS via a ribbon cable and are mounted on a plinth suspended from the sensor’s casing wall.

## 3. Results and Discussion

### 3.1. Comparison with BBCEAS

**Figure 7:**
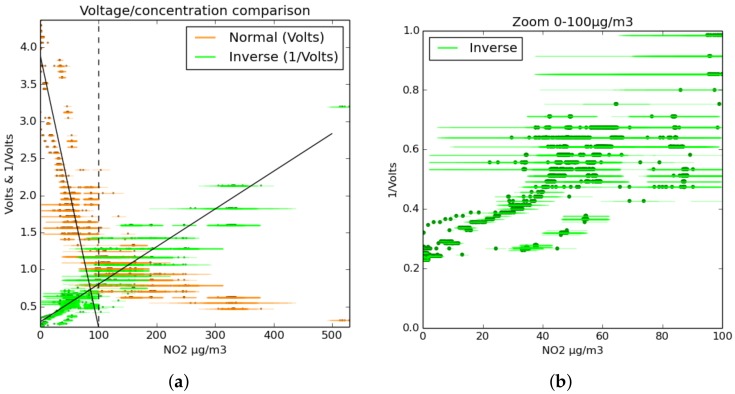
Results from the BBCEAS calibration experiment, showing both the raw output voltage and inverse voltage of the same measurement. The darker points are the data; the horizontal lines are the error ranges of the concentration. The experiment demonstrated sensitivity up to 400 $\mu g\;m^{-3}$ as shown in (**a**). Although at high concentrations, the voltages from the sensors becomes quite low, in most atmospheres, NO_2_ does not often surpass 50 ppb. For concentrations less than 100 $\mu g\;m^{-3}$, both normal and inverse voltage can be seen as approximately linear. Graph (**b**) shows the data for inverse voltage at low concentrations.

An experiment that was performed during the first part of 2015. The sensor was placed in an airtight cell with controlled temperature and humidity along with a reference Broad-band Cavity Enhanced Absorption Spectrometer system (BBCEAS), and varying concentrations of NO_2_ were pumped through the chamber, allowing a response profile to be determined, shown in Figure 7a.

In such a controlled experiment, the sensor’s resistance varied with the inverse of NO_2_ concentration over a range of 250 ppb with an $R^{2}$ of 0.97, although the error range is quite high, particularly at high concentrations where the error bars are almost as large as the quantity being measured. Still, the provisionally-suggested form of *f* in this range is:

(10) $$f_{NO2}\left(V_{OX}\right)=\frac{k_{1}}{V_{OX}}$$

where *k* are constants to be fitted. However, at low concentrations, systematic deviation from this simple formula can clearly be identified. At lower concentrations where the fractional error was higher, a linear equation also matched the data:

(11) $$f_{NO2}\left(V_{OX}\right)=k_{1}V_{OX}$$

It is clear that the equation governing the response of the sensors to varying gas concentrations is complex. In the United Kingdom, nitrogen dioxide concentrations generally stay below 100 $\mu g\;m^{-3}$, so for most cases, the sensor will respond approximately linearly. At higher concentrations, predictions using Equation (11) will start to underestimate actual NO_2_ concentration.

The results from this experiment have some important caveats. While the instrument responded strongly to changes in humidity, this response was not thoroughly examined during this experiment. Furthermore, a calibration of air quality sensors taking place under conditions where the sole control variable is the target gas can demonstrate sensitivity, but the real atmosphere is a very different environment with many potential sources of interference not represented in closed-cell experiments.

### 3.2. Calibration

**Figure 8:**
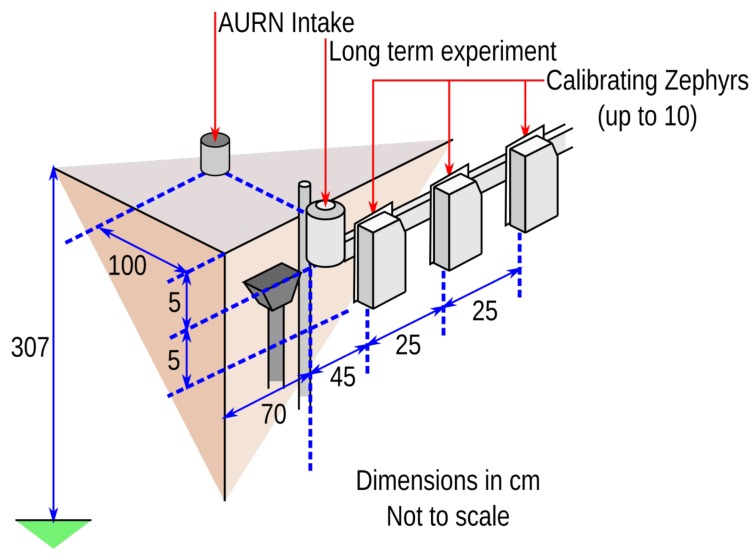
Isometric diagram of the relative positioning of Zephyr sensors near the roof of the AURN station. The lip of the roof is roughly 3 m from the ground.

For practical use of the sensors on a larger scale, an outdoor setup is used, which provides more realistic conditions. The SOGS-MOS sensors were calibrated by calculating the best fit to the reference chemiluminescence sensor in the DEFRA AURN station on Leicester University campus. The experimental setup is shown in Figure 8.

There are three different classes of instrument involved in these experiments:

- The AURN intake has an isokinetic pump that draws air down to the chemiluminescence sensors operated on behalf of DEFRA.
- The second is on a long-term SOGS-based experiment using MOS sensors. This instrument uses an older casing for pollution observing devices, but identical MOS sensor boards to the most modern Zephyr designs. This device has been out on the AURN since December 2014.
- The third group of sensor intakes belongs to MOS sensors, which are mounted on plates along a horizontal crossbar. There are ten mounting points for these sensors, roughly 25 cm apart each.

This calibration setup has issues with representativity, due to the distance between the sensor and the intake to the AURN’s own instruments combined with the high spatial variability of the target gases, their dependence on light near a structure and the irregular flow of wind over the setup. The practical consequence of these issues is discussed in Section 3.2.3.

The AURN instruments take multiple measurements every minute, but these are averaged to hourly intervals using an unknown method. For calibration, measurements are taken from the MOS sensors every 10 s, and all of those measurements that fall within 30 min of the AURN measurement times are averaged to give the comparison.

#### 3.2.1. Long-Term Experiment and Calibration Equations

**Figure 9:**
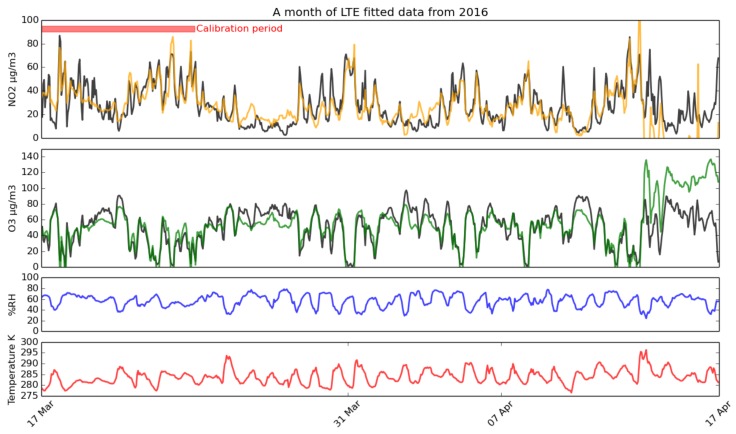
A typical month-long time series from the long-term experiment, with concentrations produced by fitting the sensor output using Equations (13) and (17) for NO_2_ (orange) and O_3_ (green) respectively to the AURN station data (black), over the calibration period highlighted in red. Temperature and humidity are also included, showing a clear diurnal cycle.

The Long-Term Experiment (LTE) has provided data for over a year, albeit with some gaps during the maintenance of the DEFRA sensor and technical issues with the SOGS prototype, giving 67% completeness. Some of the raw data from this sensor are given in Figure 9. The LTE is fitted with four MOS sensors of each type, with sensors of the same kind mounted about five centimetres apart. A data series from this sensor spanning from 5 February–16 November 2016 was analysed to determine the best equations to use with these sensors.

The simple linear Equations (10) and (11) might have proven adequate for tightly-controlled conditions, but better models are needed for the examination of sensitivity to NO_2_ and O_3_. Several equations that incorporate cross-sensitivity and variations in humidity and temperature have been developed empirically, Equations (12)–(17), and all are a linear sum of terms with the following forms for NO_2_. The *V* terms in these equations refer to voltages, which are proportional to the sensor resistance plus a constant, and the *k* terms are constants to be found.

- Linear:
  (12) $$[NO_{2}]=k_{c}+k_{1}V_{OX}+k_{2}V_{OX}RH+k_{3}V_{OX}T+k_{4}V_{O3}+k_{5}V_{O3}RH+k_{6}V_{O3}T$$
  This equation assumes that the influence of temperature and humidity is proportional to total sensor resistance and allows for ozone sensor cross-interference.
- Multiplicative linear:
  (13) $$[NO_{2}]=k_{c}+k_{1}V_{OX}+k_{2}V_{OX}RH+k_{3}V_{OX}T+k_{4}V_{OX}V_{O3}RH+k_{5}V_{OX}V_{O3}T$$
  This equation uses product terms to factor in the effect that temperature and ozone interference has on the sensor’s performance. The use of the temperature term as a multiplicative factor with the voltage was suggested by the response of CO sensors to changing temperatures in Piedrahita et al. [30].
- Inverse:
  (14) $$[NO_{2}]=k_{c}+k_{1}/V_{OX}+k_{2}/V_{OX}RH+k_{3}/V_{OX}T+k_{4}/V_{OX}V_{O3}RH+k_{5}/V_{OX}V_{O3}T$$
  This modified equation uses inverse terms in an attempt to more accurately model the curve of the sensor’s response over a greater range.
- Inverse NO_2_, Linear O_3_ multiplicative:
  (15) $$[NO_{2}]=k_{c}+k_{1}/V_{OX}+k_{2}/V_{OX}RH+k_{3}/V_{OX}T+k_{4}/V_{OX}V_{O3}RH+k_{5}/V_{OX}V_{O3}T$$
  An alternative to the inverse equation that maintains linear terms on the ozone voltage is abbreviated as InvNO2LinO3.

The calibration equations for NO_2_ account for temperature, relative humidity and ozone interference in sensor response. The strong dependence of sensor resistance on temperature and relative humidity changes has previously been described in the Materials section. Consequently, a temperature and relative humidity measurement is taken for each voltage measurement so they can be corrected for. The ozone equation is:

- Linear:
  (16) $$[O_{3}]=k_{c}+k_{1}V_{O3}+k_{2}V_{O3}RH+k_{3}V_{O3}T$$
  This simple equation keeps the multiplicative terms for temperature and humidity.
- Inverse:
  (17) $$[O_{3}]=k_{c}+k_{1}/V_{O3}+k_{2}V_{O3}RH+k_{3}/V_{O3}T$$
  an ozone equation with inverted voltage terms.

To evaluate which equation gives the best results, a six-step process was used over the LTE time series:

1. The AURN and MOS sensor time series was sliced into sections between six and seven days long when data were available, contracting to a minimum of six days where there were gaps in the data, at hourly offsets.
2. A linear fit was taken using the calibration equation over every one of these periods, giving an array of fit coefficients.
3. Each set of these coefficients was used to predict gas levels for the entire time series, producing an array of predicted time series.
4. Using a similar moving window over each predicted data series, statistical functions measuring the goodness of fit were calculated. These functions are the Pearson correlation coefficient between prediction and reference (Equation (9)), residual standard error (Equation (7)) and residual fractional error (Equation (8)). Each application of this method to a prediction gives an error time series, which represents how good the fit is at a particular time. At the end of this process, there is one error time series for each prediction.
5. The timestamps of the error time series were shifted so that they aligned over the week at which the calibration was taken. Thus, at time = zero, the standard error array for a particular time series gives the error over the same period at which the calibration that produced it took place.
6. Finally, all of the error time series were split into day-lengths, and an average of all of them over each day was taken to give the statistical errors of the sensor’s prediction as a function of the time from the calibration. The standard error of the data in these day-long bins is a representation of the variation in quality of fits at predicting data at that particular time. The gaps in the original data are averaged over the entire length of this output, making it continuous.

This technique eliminates possible bias from choosing an arbitrary calibration period and predicting the rest of the time series from it. It also gives a sense not just of the reliability of a sensor’s data a given time from calibration, but also for the variation in quality that calibrations can have depending on the particular atmospheric conditions at the time.

**Figure 10:**
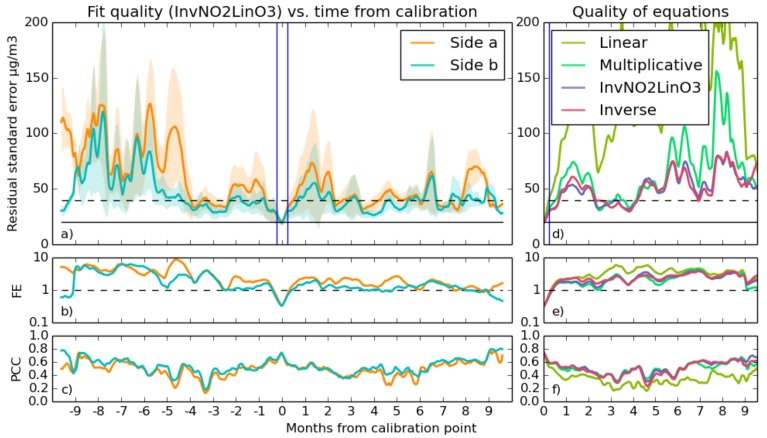
Long-term experiment performance for a pair of sensor elements, calibrated for NO_2_ using the inverse NO_2_ linear O_3_ equation. Shown in the largest graph (**a**) is the mean rolling Residual Standard Error (RSE) for the pair, with the shaded region showing the range within which 95% of the fits were performed. (**b**) is a graph of logarithmic Fractional Error (FE), and (**c**) shows the rolling fit Pearson Correlation Coefficient (PCC) against time From Calibration (tFC). In the right graphs is a comparison of different equations with the sensor pair results and positive and negative times averaged together. The blue line delineates the calibration period; the horizontal solid black line indicates the target accuracy of 20 $\mu g\;m^{-3}$; and the dotted black line is twice this. On the right in graphs (**d**–**f**) are a comparison of the performance of the four different equations referenced in Section 3.2.1. Each data point in these graphs is an average of the absolute time from calibration and of both sensors.

**Figure 11:**
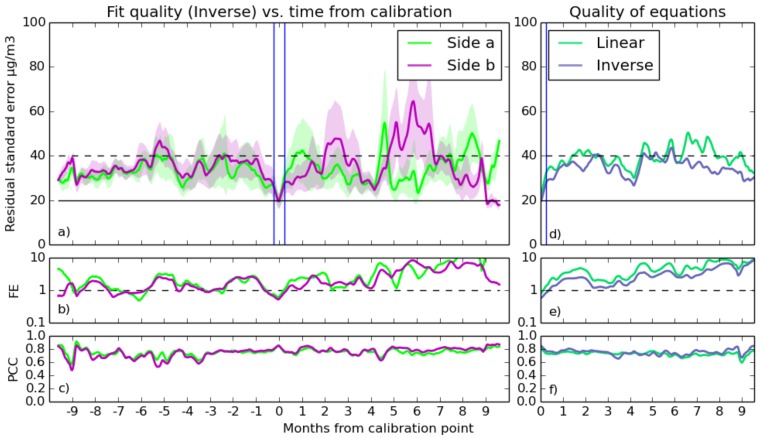
As Figure 10, but for ozone.

The statistics of fit quality are plotted in Figure 10 and Figure 11, which show in detail the best fit equation, as well as comparing the different equations in terms of quality.

The RSE/tFC graph at the top left shows some features in common for both of the proximate sensors, in both NO_2_ and O_3_. The large instability in earlier time periods in the NO_2_ graph indicates that fits taken toward the end of the year were somehow not suited for measurements at the year beginning. The implication is that the calibration environment has caused these anomalies, and the common, but varying, intensity of the inaccuracy suggest that interfering gases are present, to which one of the sensors is slightly more sensitive than the other.

PCC starts at roughly 0.8 for NO_2_ and 0.83 for O_3_, but in the case of NO_2_, it decreases steadily and holds between 0.4 and 0.65, reaching its lowest point 2–3 months from the calibration time on the PCC/tFC graph. One possible explanation for a drift on this time scale is that seasonal variation in background gasses causes the sensor’s output signal to drift on such a time scale. This effect could also be caused by variation in heater element temperature caused by natural degradation over time, which is an effect documented by Masson et al. [15].

The fractional error graphs of both sensors shows a striking increase in FE (up to ten) further than about four months away from the time of calibration, albeit at different times in Figure 10 and Figure 11. These results are obviously unacceptable and coincide with increases in absolute RSE that suggest that the error is due to a limitation in the sensors or the calibration process, rather than the high fractional error being a mere consequence of the low concentration of the target gasses in certain parts of the year.

As the comparison of different equations shows, involving inverse terms allows the sensor’s non-linearity to be more accurately modelled. Models that do not use inverse terms suffer from occasional bursts of inaccuracy, and using equations with multiplicative scaling of temperature terms with voltage greatly improves accuracy compared to purely linear models.

It is clear that the fits generally have the best quality during the week they were taken (time = 0 on the above figures). Fits become less reliable further out, with the RSE/tFC graph showing typical accuracies that are comparable to the typical concentrations of the target gases in the atmosphere. The FE/tFC graph underlines this fact; only in the weeks surrounding calibration is the sensor error consistently smaller than the magnitude of the reference instrument’s reading for NO_2_ and O_3_, suggesting that our current methods are giving unreliable results.

However, there is a hint in this data that we can do better. The better end of the standard deviation region (shaded area on the RSE/tFC graph) hovers at much better accuracy. This is the standard deviation of the quality of all of the individual fits, and some fits are much better than others. If the quality of these fits is due to some environmental condition during calibration, and they are consistently good throughout the year, it might be possible to test for these conditions and discard the poorer fits.

#### 3.2.2. Selecting Good Fits

To determine the quality of the coefficients produced during a week-long calibration period, the sensor is kept at the calibration site for a second week (the validation period), and using the three tests, its output during that time is compared to that of the reference. There are arbitrary thresholds of quality for each test: more stringent thresholds may improve quality, but will also lead to more fits being rejected. Thresholds for this analysis were:

- With RSE, a fit qualifies if its standard error is below 20 $\mu g\;m^{-3}$, in line with the accuracy goals.
- For FE, the test is passed if the fractional error is below one. This is a fairly loose constraint, but during some calibrations, the predicted values, which are reasonable during the calibration period, start to deviate drastically during the validation period. This test will filter out those cases.
- There were several thresholds for correlation used, as the different gas fits typically were capable of differing levels of quality. Values of 0.6, 0.7 or 0.8 are all compared for both gases.

**Figure 12:**
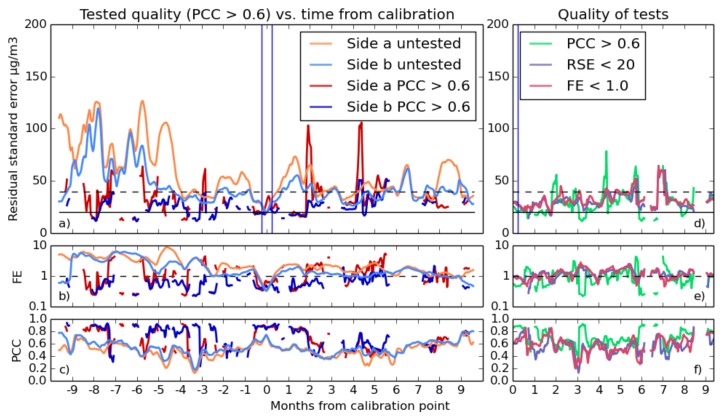
(**a**–**c**) Comparison between sensor data for NO_2_ without goodness testing (light) and with (dark). Both Sensors a and b are plotted. Since fewer fits are used, error due to averaging is higher, and there are both gaps and alarming spikes in the RSE in graph (**a**). However, testing for fractional error improves both FE and correlation. (**d**–**f**) shows the effect using various tests has on the statistics of the data, averaging the absolute time from calibration and over both sensors.

**Figure 13:**
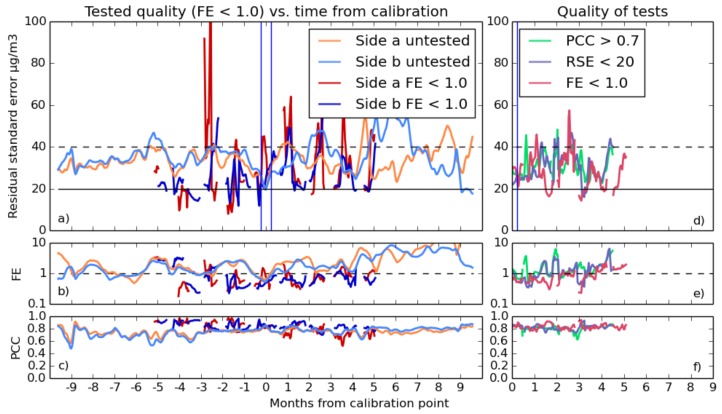
As for Figure 12, but with O_3_.

For the long-term experiment calculations, the test is applied between Steps 2 and 3. Often large numbers of predictions had to be discarded, and if at Stage 6 there were less than five time series to average together, their characteristics were omitted from the final result. The results of the selection process are shown in Figure 12 and Figure 13.

The figures show mixed results; while the occasional increases in error might seem paradoxical, note that the test is applied one week after the fit is produced. Averaging sensor performance over the entire experimental period allows us to compare different tests over a period of 20 months. Table 2 shows the mean fit quality for all of the different testing parameters. Averaging the sensor performance variables over one month shows possible results from a short-term deployment, as in Table 3.

Generally using tests reduces errors substantially, at a cost of risking a longer wait to get a good calibration. Discarding all fits that do not meet a correlation coefficient threshold appears to be the most reliable means of decreasing sensor error, and it is this test (though with different thresholds) that is shown in Figure 12 and Figure 13. The sensor data are now frequently within specification, but at a cost of occasionally giving worse results and reducing the number of valid fits. In practice, this can mean that more than two weeks must be spent to get a good, validated calibration.

The best goodness test to use for NO_2_ would appear at first glance to be correlation greater than 0.8; however, this also results in only about one in nine calibrations actually being suitable for use. A correlation better than 0.6 is more practical, providing a moderate improvement in results while discarding one half to two thirds of calibrations.

The situation is more nuanced with O_3_. The tests involving correlation actually make the situation worse, and RSE is universally “shifted” through discarding bad fits. However, a test involving discarding fits that give less than 1.0 fractional error serves to reduce this value, improves correlation and is the only test able to do this. Generally, O_3_ does not improve much from being selective with calibrations.

For both target gasses, introducing selective calibrations constrains the effective range of results. Only data reasonably close (within four or five months) to the sensor’s calibration time produced good results, which implies a limit on the amount of time a sensor can be redeployed between calibration periods.

#### 3.2.3. Calibration and Validation Times

For the purposes of deployment in the field, the sensors are calibrated at the AURN station for a period of two weeks, with one week being the source of the calibration fit and the other being a period over which the fit can be evaluated to see if the sensor’s output has the desired accuracy (95% confidence within 20 $\mu g\;m^{-3}$). These time periods were chosen principally for convenience and because with hourly measurements, this amount of time permits a reasonable fit to be found.

### 3.3. Warm-Up Time

The time taken for the MOS sensors to reach equilibrium was investigated. There are many practical reasons that sensors deployed in the field may have their power interrupted momentarily. If there is no battery backup, installing the sensors in a car means disconnecting their power supply for up to five minutes. During one mobile campaign, a sensor, equipped with a transceiver, experienced periodic brownouts when the transceiver tried to connect to the GSM network to deliver data every six minutes or so. This caused periodic drops in supply voltage to the MOS sensor boards, with corresponding blips in the data from them.

#### 3.3.1. Experimental Method

A MOS instrument was placed in a closed plastic container in a dark corner of an air conditioned lab for three days to approach a stable atmosphere. The instrument’s power supply was then interrupted for various durations and the sensor response observed to determine how long it would take for them to return to a baseline. The fans of the unit were operated while it was active.

During the experiment, the box was kept in the dark with the sensor (and hence, the sensor heating elements) powered up. At various points, the power to the sensor was interrupted, and after a specific period of time, the power was restored. The movement over time of the raw voltages from the sensors in the static atmosphere after their power was interrupted is the crucial feature of the experiment, compared with the voltage they would settle to after a prolonged period of inactivity.

**Figure 14:**
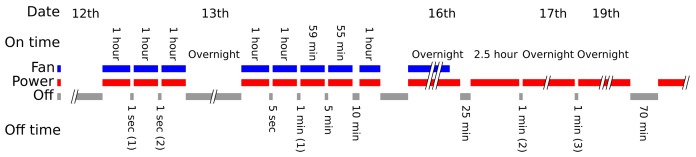
A timeline of the power supply interruption experiment, which took place over multiple days in December 2016. The labels for the periods during which the power supply was interrupted are referenced in the Results section.

**Figure 15:**
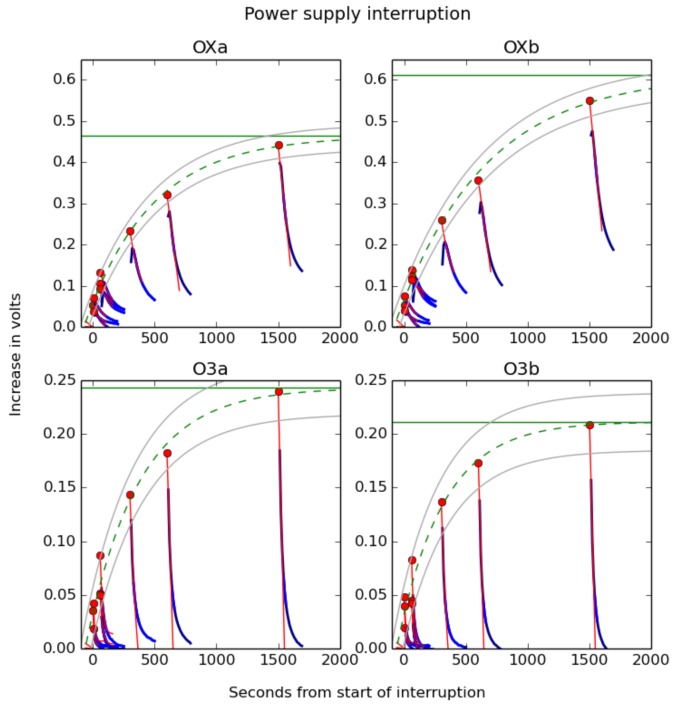
Fitted curves after power supply interruption for four different sensors, two each of oxidising (OXa and OXb) and ozone (O3a and O3b), mounted in the same enclosure. The blue curves are the time-shifted raw voltage curves; the red lines are hysteresis-compensated voltage curves with points at the time at which the power supply was reconnected; the dashed green line is an exponential curve fitted to these points showing the likely voltage curve when the power is disconnected and the sensor is cooling; and the solid green line is the unpowered baseline voltage inferred from this fit. The grey curves above and below the exponential fit are the 95% confidence interval for it.

The timeline of the experiment is shown in Figure 14. The voltage responses from the four sensor elements in question are shown in Figure 15.

#### 3.3.2. Discussion

The voltages from the sensors generally fit the characteristic form shown in Figure 3 after the power connection was restored, although immediately after the supply was reconnected, the oxidizing gas sensors showed increasing voltages for a couple of seconds before they began decaying down to the baseline level, indicating sensor hysteresis that was more pronounced for the oxidizing gas sensors than the ozone sensors. This effect was cancelled out by fitting exponential curves to each individual decay curve and taking the intercept at the point when the power was restored.

The magnitude of the voltage jump after the power supply interruption, once hysteresis is compensated for, follows the exponential decay law described in Equation (6). The 95% confidence intervals for the fits to the exponential curves range between 0.029 V and 0.341 V between different sensors, although the fractional error is much higher for points close to time when the power was restored, and the fit gets much better further away.

Different magnitudes of response ($k_{a}$ and $k_{c}$) were observed for different sensors, despite the ambient conditions being the same. This points to the inherent differences between sensors due to the manufacturing process. However, this experiment proves that the exponential decay constant during different warm-up periods is the same even with different baseline voltages and can thus be compensated for.

By fitting exponential curves to the data after power supply interruption, it is possible to compensate (at least superficially) for the warm-up effect. However, while it is likely that the sensor elements experience a dulled sensitivity to target gases during this period, this hypothesis could not be tested during this experiment.

### 3.4. Airflow

**Figure 16:**
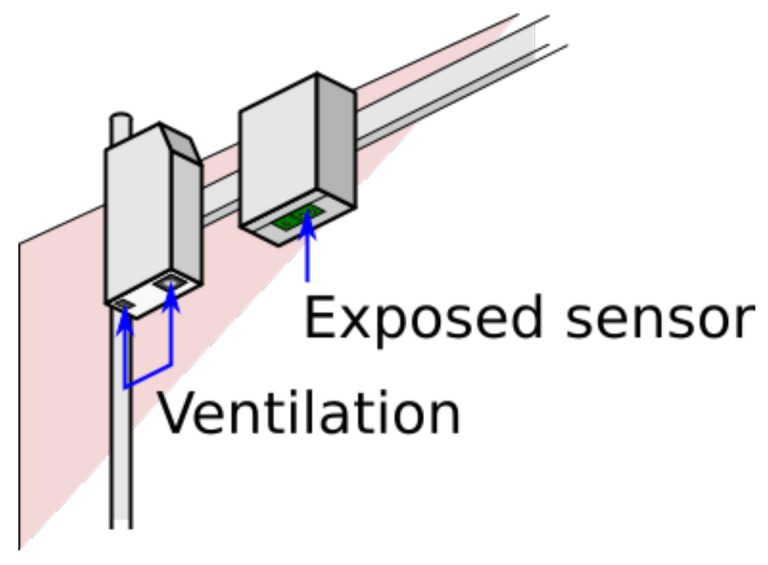
Experimental setup for investigating aspirated (on the left) vs. passive sampling (right side), on the AURN station.

To investigate the effects of using fans to cycle air through a vented enclosure, compared to an enclosure that carries the sensor elements on the outside facing the atmosphere, two prototype instruments with identical electronic configuration were placed in different casings. One of these enclosures was equipped with fans; the other had a cutaway that allowed the surface of the sensors to be exposed to the air beneath the casing. These were connected to the same power supply in parallel and installed next to each other on the AURN station for calibration over a period of two weeks, as shown in Figure 16. The sensors were both set to take measurements every five seconds. In the active casing, a measurement was immediately preceded with a second-long pulse of the fans.

#### Discussion

**Figure 17:**
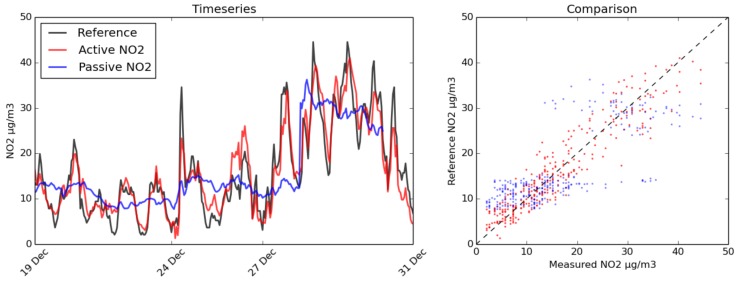
The effectiveness of aspiration (red) in comparison with the effectiveness of passive airflow for a sensor exposed to the elements (blue). The AURN (black) was used as a reference for actual NO_2_ concentrations. On the left is a time series; on the right is a scatter plot of the same data demonstrating the diminished sensitivity with passive airflow.

The marked difference in sensitivity for this instrument when fans are not in place is clear from Figure 17. While it should be possible to design a passively ventilated casing that would be more sensitive, for these particular enclosures, the lack of fans discounts the use of the sensors unless a day-long integration time is acceptable.

### 3.5. Variability between Sensors: Environment or Manufacturing?

As discussed in Section 1.1, MOS gas sensors exhibit a high degree of individuality between individual specimens by their very nature. However, when using these sensors to measure NOx, the fact that these species tend to vary in concentration on a scale of seconds and centimetres is a complicating factor in calibrating them to say the least. Due to the architecture of the MOS instrument board used with SOGS, it can be shown that two sensors separated by a centimetre and shielded from sunlight produce good correlation in their output.

**Figure 18:**
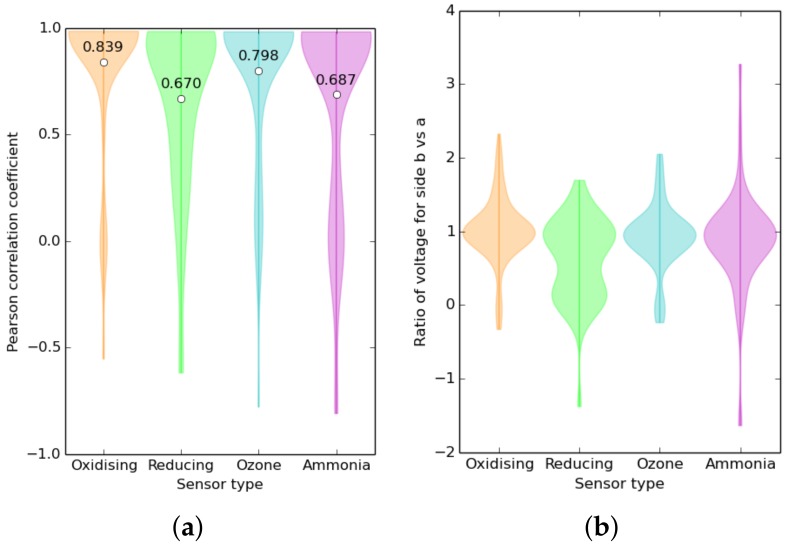
(**a**) Violin plot of Pearson correlation between the a and b sensors. The mean correlation between sensor pairs is marked with a white dot and labelled. (**b**) Violin plot of the distribution function of the gradient of a linear fit of Side a to Side b. This plot essentially shows the distribution of the average ratio of pairs of sensors to each other. The output voltage of the sensor electronics in this experiment is linearly proportional to the element resistance.

One hundred and twenty one sets of data from many different experiments across a number of sensors were collated, and statistics were taken of the relationship between the four different pairs of sensors on each board (see Figure 5). The data series ranged up to a year in length, but data series less than a day long were omitted from this calculation to minimize the warm-up effect. The median experiment length was 7.9 days, and most of them represented either two-week calibrations or week-long deployments. The statistics in aggregate can be seen in Figure 18.

The character of the variation between sensors is interesting. For example, the gain of the reducing gas sensors varies markedly with a large clustering of the gradient around zero, and this corresponds to a lower correlation coefficient, which likely indicates that either the electronics were saturated when driving with these sensors or a proportion of them were faulty. The NH3 sensors also exhibited highly variable gain.

## 4. Conclusions

The techniques described in this paper allow relatively simple models to give favourable performance, provided calibrations are carefully selected. Without the use of goodness tests, the average 95% confidence interval for NO_2_ scored between 32.6 $\mu g\;m^{-3}$ and 37.5 $\mu g\;m^{-3}$ for the pair of sensors and O_3_ between 30.5 $\mu g\;m^{-3}$ and 28.4 $\mu g\;m^{-3}$ (see Table 2), with wide variation depending on when the calibration was taken. This is up to 5.58 $\mu g\;m^{-3}$ worse for NO_2_ and 6.1 $\mu g\;m^{-3}$ worse for O_3_ than the results obtained by Piedrahita et al., although the calibration method is not directly comparable. However, using a moderately stringent criteria for discarding `’bad” calibrations, these numbers can be substantially improved in the case of NO_2_ to 20.0 $\mu g\;m^{-3}$ and 20.3 $\mu g\;m^{-3}$ (see Table 3). The ozone does not experience an improvement in absolute confidence from using goodness tests, but fractional error can be cut by nearly half.

Even with the use of these techniques, the phenomenon of sensor drift was not corrected for here, and it truncated trustworthy results to data to within four months from calibration. This is a similar scale as has been reported by Spinelle et al. and Masson et al. and somewhat of an improvement over the results described in the former. An analytical approach to counteracting this drift might be “merging calibrations”, where a sensor is calibrated at the start and end of a four-month campaign, and the coefficients gradually change from one end of the experiment run to the other.

There are numerous paths for further research involving these sensors. First, while the response curve of MOS sensors under controlled conditions where only one variable changes has been well characterized by the manufacturers, by other researchers and by this group under controlled conditions, their response to multiple variables is still not well understood and lies at the heart of the question of their reliability. More exhaustive testing should be undertaken in an artificial atmosphere and with different heater element temperatures in an attempt to understand the role of interfering gases better.

Further to this, it is clear that there are two major factors in the longevity of a sensor’s calibration. The first is the natural degradation of the heater element, which becomes hotter over prolonged use [15] and causes the sensor’s response profile to vary. The second is the effect of slowly-varying interfering gases, which over the course of months shift the sensor’s baseline. The first problem may have an engineering solution, but the second will involve taking the results of the tests in an artificial atmosphere, identifying the most critical species and either measuring or possibly modelling their likely concentrations during deployments.

The calibration setup for the sensors is not currently optimal, but the strong correlation of sensor voltages within the same housing suggests a solution in ensuring that the same packet of air reaches the MOS sensors as the reference instrument, in as little time as possible. The rapid rate at which NOx compounds evolve and the sensitivity of the Leighton system to sunlight means that the representativity of the calibration environment is even more critical than it would be for more specific sensors.

The results obtained during the course of this work suggest that metal oxide semiconductor gas sensors have sufficient sensitivity and responsiveness to be useful in urban air quality, well outside their original role in industrial safety systems. While challenges remain in the use of such sensors in long-term deployments, their low cost makes them well suited to campaigns with month-long time scales in urban environments or as mobile sensors.

## Figures and Tables

**Table 1:** Summary of sensors utilised. Due to the individuality of each gas sensor, the resolution of the measurements could not be provided by the manufacturer.

Parameter Measured	Sensor Part Number	Method of Detection	Gas Detected and Detection limits	Resolution
Reducing Gases	SGX Sensortech MICS-4514	redox reaction	CO: 1–1000 ppm	N/A
			NH_3_: 1–500 ppm	
			C_2_H_5_OH: 10–500 ppm	
			H_2_: 1–1000 ppm	
			CH_4_: >1000 ppm	
Oxidising Gases	SGX Sensortech	redox reaction	NO_2_: 0.05–10 ppm	N/A
	MICS-4514		H_2_: 1–1000 ppm	
O_3_	SGX Sensortech	redox reaction	10–1000 ppb	N/A
	MICS-2614			
NH_3_	SGX Sensortech MICS-5914	redox reaction	NH_3_: 1-500 ppm	N/A
			C_2_H_5_OH: 10–500 ppm	
			H_2_: 1–1000 ppm	
			C_3_H_8_: >1000 ppm	
			C_2_H_8_(CH_4_)_2_: >1000 ppm	
Temperature and Relative Humidity	GE Measurement and Control CC2D25	Polyimide capacitance	Temperature: −40–125 °C Relative humidity: 0–100%	±0.3 °C 2%

**Table 2:** Table of means and 95% confidence intervals for all goodness tests across the entire span of data, including data compiled without tests for reference. Rows discussed in the text are highlighted. For correlation, a higher mean score is better. For fractional error and residual standard error, a lower mean is better. Low 95% confidence intervals (2SD) are better all round.

Gas	Metric	Side	Untested		PCC > 0.6		PCC > 0.7		PCC > 0.8		RSE < 20		FE < 1.0	
			Mean	2SD	Mean	2SD	Mean	2SD	Mean	2SD	Mean	2SD	Mean	2SD
NO_2_	RSE	**a**	57.2	50.1	32.5	33.5	24.7	13.1	20.3	9.3	34.5	31.1	35.0	29.6
		**b**	44.2	33.7	25.7	16.5	22.2	11.4	20.4	10.1	29.0	16.3	28.4	14.3
	FE	**a**	2.60	3.28	1.38	2.20	0.77	1.05	0.63	1.05	1.47	1.86	1.51	1.91
		**b**	1.98	2.94	0.67	0.73	0.59	0.69	0.44	0.27	1.01	1.08	0.94	1.07
	PCC	**a**	0.49	0.23	0.68	0.33	0.73	0.37	0.87	0.09	0.57	0.33	0.57	0.33
		**b**	0.54	0.22	0.71	0.35	0.72	0.38	0.85	0.14	0.54	0.46	0.60	0.35
	Used	**a**	100%		54%		15%		12%		75%		82%	
		**b**	100%		60%		24%		11%		74%		74%	
O_3_	RSE	**a**	33.6	10.9	34.4	32.4	32.6	29.0	35.4	34.6	32.6	29.4	31.6	31.9
		**b**	35.5	15.8	32.4	22.4	30.3	26.8	27.1	17.5	31.0	28.8	26.4	18.3
	FE	**a**	3.62	8.79	3.09	13.5	3.31	15.3	3.42	14.7	3.12	14.8	0.89	1.27
		**b**	2.37	3.67	2.01	3.53	2.13	3.92	1.52	3.40	1.94	4.10	0.91	1.52
	PCC	**a**	0.75	0.09	0.78	0.18	0.81	0.15	0.81	0.15	0.80	0.18	0.80	0.19
		**b**	0.75	0.14	0.81	0.14	0.82	0.14	0.84	0.11	0.83	0.12	0.85	0.12
	Used	**a**	100%		79%		81%		58%		75%		29%	
		**b**	100%		82%		78%		56%		75%		42%	

**Table 3:** Table of means and 95% confidence intervals for goodness tests across a month-long span. The shorter period results in higher precision and accuracy. For correlation, a higher mean score is better. For FE and RSE, a lower mean is better. Low 95% confidence intervals (2SD) are better all round.

Gas	Metric	Side	Untested		PCC > 0.6		PCC > 0.7		PCC > 0.8		RSE < 20		FE < 1.0	
			Mean	2SD	Mean	2SD	Mean	2SD	Mean	2SD	Mean	2SD	Mean	2SD
NO_2_	RSE	**a**	37.5	22.6	24.4	9.69	20.5	6.37	20.0	5.43	28.9	12.1	28.5	10.1
		**b**	32.6	14.0	19.9	5.09	19.3	7.37	20.3	4.31	23.4	8.70	22.7	7.77
	FE	**a**	1.39	1.34	0.80	1.35	0.49	0.36	0.50	0.38	1.16	1.03	0.99	0.82
		**b**	1.06	0.88	0.66	1.01	0.41	0.21	0.46	0.28	0.62	0.44	0.59	0.39
	PCC	**a**	0.57	0.13	0.85	0.14	0.89	0.04	0.89	0.02	0.64	0.28	0.64	0.30
		**b**	0.59	0.14	0.82	0.27	0.86	0.31	0.89	0.04	0.69	0.29	0.67	0.37
	Used	**a**	100%		58%		50%		50%		75%		83%	
		**b**	100%		77%		58%		45%		87%		85%	
O_3_	RSE	**a**	30.5	12.8	28.9	16.6	27.0	10.9	27.9	17.0	23.9	7.64	33.3	22.1
		**b**	28.4	8.21	29.8	17.3	27.7	18.8	25.4	13.0	25.4	9.53	26.8	12.0
	FE	**a**	1.41	1.26	0.81	1.11	0.83	0.91	1.28	1.83	0.76	0.83	0.80	0.85
		**b**	1.04	0.77	1.21	2.15	1.46	3.13	0.81	1.07	0.53	0.33	0.53	0.32
	PCC	**a**	0.77	0.07	0.83	0.12	0.82	0.12	0.82	0.14	0.84	0.10	0.80	0.12
		**b**	0.78	0.06	0.81	0.15	0.82	0.20	0.84	0.10	0.83	0.08	0.84	0.06
	Used	**a**	100%		90%		88%		83%		73%		50%	
		**b**	100%		91%		77%		75%		93%		80%	

## Abbreviations

The following abbreviations are used in this manuscript:

OMI	Ozone Monitoring Instrument
ANDI	Atmospheric Nitrogen Dioxide Imager
AURN	Automatic Urban/Rural Network
DEFRA	Department for the Environment, Food and Rural Affairs
MOS	Metal Oxide Semiconductor
LTE	Long-Term Experiment
FE	Fractional Error
RSE	Residual Standard Error
PCC	Pearson Correlation Coefficient
tFC	Time From Calibration
InvNO_2_LinO_3_	Inverse NO_2_, Linear O_3_ equation
